# Association of Prenatal Ibuprofen Exposure with Birth Weight and Gestational Age: A Population-Based Sibling Study

**DOI:** 10.1371/journal.pone.0166971

**Published:** 2016-12-09

**Authors:** Kateřina Nezvalová-Henriksen, Mollie Wood, Olav Spigset, Hedvig Nordeng

**Affiliations:** 1 PharmacoEpidemiology and Drug Safety Research Group, School of Pharmacy, University of Oslo, Oslo, Norway; 2 Department of Clinical Pharmacology, St Olav’s University Hospital, Trondheim, Norway; 3 Department of Laboratory Medicine, Children’s and Women’s Health, Norwegian University of Science and Technology, Trondheim, Norway; 4 Domain for Mental and Physical Health, Norwegian Institute of Public Health, Oslo, Norway; Iran University of Medical Sciences, ISLAMIC REPUBLIC OF IRAN

## Abstract

**Objectives:**

Three studies so far have investigated the effect of prenatal non-steroidal anti-inflammatory drug (NSAID) exposure on birth weight and gestational age. The aim in this study was to evaluate the association of prenatal ibuprofen with birth weight and gestational age at birth, using a sibling design in an attempt to adjust for the possibility of familial confounding.

**Design:**

Using data from the Norwegian Mother and Child Cohort Study (MoBa) and the Medical Birth Registry of Norway (MBRN), we identified 28 597 siblings, of whom 1080 were prenatally exposed to ibuprofen and 26 824 were not exposed to any NSAID. Random and fixed effects models with propensity score adjustment were used to evaluate the effects of ibuprofen exposure on birth weight and gestational age.

**Results:**

Ibuprofen exposure during the first trimester was associated with a decrease in birth weight of 79 grams (95% confidence interval -133 to -25 grams). In contrast, second and/or third trimester exposure, and duration of exposure had no impact on the effect estimates. We found no association between ibuprofen exposure and gestational age at birth.

**Conclusions:**

Our results suggest that prenatal exposure to ibuprofen during the first trimester is associated with a slight decrease in birth weight. The association does not seem to be attributable to shared genetics and family environment, and could be explained by either exposure to ibuprofen, or to non-shared confounding between pregnancies.

## Introduction

The prevalence of non-steroidal anti-inflammatory drug (NSAID) use during pregnancy ranges between 5% and 17%.[1–7] Most studies on prenatal NSAID exposure focus on spontaneous abortion [8–14], congenital malformations[4, 15–23], and neonatal intraventricular haemorrhage (IVH).[22, 24, 25] Several of these studies find increased risks for negative pregnancy outcomes after exposure in early and late pregnancy. Therefore most clinical guidelines today recommend caution in use in the first and third trimester.[6] Despite the fact that infants born prematurely and having low birth weight are at an increased risk of mortality and morbidity only three studies have investigated the effect of prenatal NSAID exposure on birth weight and gestational age so far.[4, 10, 13] Among these studies two did not find any increased risk of low birth weight or premature delivery.[10, 13] However, both these studies were underpowered and only included 1742 and 145 exposed infants respectively. The third study, a previous study from our group analyzing data from the Norwegian Mother and Child Cohort included over 6500 infants exposed until gestational week 29, and that study found an association between prenatal ibuprofen exposure and low birth weight (OR 1.4; 95%CI 1.1 to 1.6), but not preterm birth (OR 1.1; 95%CI 1.0 to 1.3).[4]

Since thousands of pregnant women use NSAIDs worldwide, and most commonly ibuprofen, even a small effect on birth weight or gestational age could have a significant impact on public health. Although we in our previous study[4] had sufficient statistical power and a vast array of information on confounding to adjust for, we could not account for confounding by genetics and familial factors. It is well known that genetics, both parental and fetal, are risk factors for low birth weight and to a lesser extent reduced gestational age[26, 27], and there is a possibility that our previous findings were influenced by these factors.

The aim of the present study was to reevaluate the association of prenatal ibuprofen exposure with birth weight and gestational age using a sibling design. Such a design allows for adjustment of genetic and familial confounding factors that are shared between siblings, because siblings share on average 50% of their genes and to a large degree share family environment.[28] In order to adjust for confounding factors that are shared by siblings, we have applied a fixed effects model, which includes sibling clusters of all sizes rather than only pairs.[29] Comparing the outcomes of siblings with concordant and discordant ibuprofen exposure status allows for the efficient control of confounders that are shared between siblings. We used a random (estimates comparable to an unrelated cohort design) and fixed (estimates for the difference between exposure-discordant siblings) effects modelling strategy combined with propensity score methods to adjust for both measured and unmeasured confounders.

## Methods

### Study design

This population-based sibling study was based on The Norwegian Mother and Child Cohort Study (MoBa) and The Medical Birth Registry of Norway (MBRN). MoBa and MBRN were linked via the 11-digit maternal identification number assigned to every resident of Norway.

MoBa is an ongoing observational prospective cohort study conducted by the Norwegian Institute of Public Health (NIPH).[30] The principal objective of MoBa is to evaluate the effect of a vast array of prenatal exposures on the health of the child.

All pregnant women living in Norway who gave birth between 1999 and 2008 were invited to participate in MoBa. There were no exclusion criteria, and the participation rate was 40.6%.[30, 31] Information on maternal medical, socio-demographic, and lifestyle characteristics before and during pregnancy was obtained from two self-administered questionnaires. The first questionnaire was completed at gestational week 17, Qw17, covering the period between six months prior to pregnancy and gestational week 18. The second questionnaire was completed at gestational week 30, Qw30, and covered the second and third trimesters of pregnancy. The response rates were 94.9% (Qw17) and 91.0% (Qw30).[32] The current study is based on version eight of the quality-assured data files released for research in 2014.

The MBRN includes all pregnancy outcomes in Norway after the 12th gestational week. Information on maternal health both prior to and during pregnancy, the course of pregnancy and pregnancy complications, delivery and postpartum complications and interventions, and the health of the neonate is available from standardised mandatory forms completed by midwives and obstetricians and/or gynaecologists at each delivery and from antepartum obstetric records completed by general physicians, gynaecologists, or midwives throughout pregnancy.

### Study population

**Fig 1:**
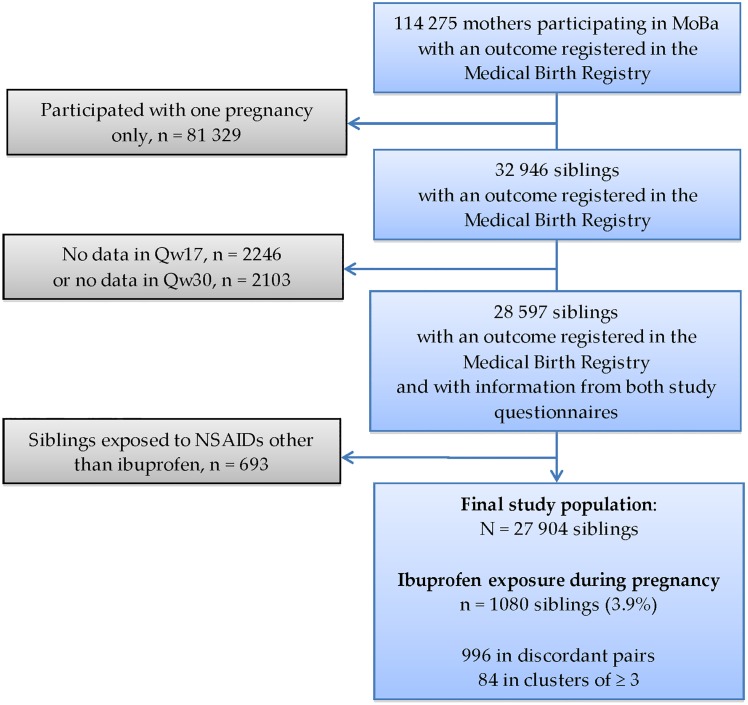
Population flow diagram. MoBa: Norwegian Mother and Child Cohort Study. NSAIDs: Nonsteroidal antiinflammatory drugs.

Of the initial population of 114 275 pregnant women, 32 946 participated more than once. After excluding multiple pregnancies, pregnancies lacking information in any of the two study questionnaires and siblings exposed to other NSAIDs than ibuprofen, a total of 27 904 siblings were included in the final study population. The final study sample included 1080 siblings (3.9%) exposed to ibuprofen during pregnancy; of these 996 belonged to a sibling pair and 84 were in clusters of three or more. The remaining 26 824 siblings (96.1%) were unexposed to any NSAID. Women included in our study sample had equivalent socio-demographic characteristics to those not included. A flow chart of the study population is shown in Fig 1.

The mothers of the siblings included in the final study sample displayed similar socio-demographic characteristics compared with the mothers in the initial MoBa cohort participating with one child only. However, a statistically higher proportion of the mothers in the sibling study sample had a medical diagnosis before but not during pregnancy (56% in the sibling sample compared with 44% in the cohort).

### Ibuprofen exposure during pregnancy

Information on the timing and duration of ibuprofen exposure was available from the two MoBa questionnaires answered during pregnancy (Qw17 and Qw30). The questionnaires were designed to increase recall and several indications where analgesics could be applied were specifically named: musculoskeletal pain (acute), inflammatory musculoskeletal conditions (both chronic and acute), infectious diseases (acute), neuromuscular inflammatory conditions (acute), and inflammatory conditions accompanied by symptoms of pain. An open-ended question about medications use was also included. For each indication, medication use in the following time periods could be specified: gestational weeks 0 to 4, 5 to 8, 9 to 12, and 13+ (Qw17) and weeks 13 to 16, 17 to 20, 21 to 24, 25 to 28, and 29+ (Qw30). Drug exposure was classified and grouped according to the Anatomical Therapeutic Chemical (ATC) Classification System developed by the World Health Organization.[33] Ibuprofen exposure was defined as exposure to a drug belonging to ATC class M01A E01. The following explanatory exposure variables were then created: ‘ibuprofen exposure anytime during pregnancy’, ‘ibuprofen exposure during the first trimester only’, ‘ibuprofen exposure during the second and/or third trimesters only’–these exposure variables addressed the effect of timing of exposure on outcome; ‘ibuprofen exposure during any one trimester’ and ‘ibuprofen exposure during two or more trimesters–these exposure variables addressed the effect of duration of exposure on outcome.

### Birth weight and gestational age

The outcome variables in our study, birth weight in grams and gestational age in days were derived from the MBRN. Birth weights outside 3.5 standard deviations from the gender specific mean at each pregnancy week (0.5%) and gestational ages exceeding 44 weeks (0.9%) were recoded as missing. We tested the intra-class correlation for gestational age and birth weight to evaluate similarity within the sibling clusters.

### Potential confounding variables

The following measured medically plausible confounding variables were included in the propensity score that was used as the adjusting variable: maternal age, parity, pre-pregnancy Body Mass Index (BMI), education, smoking throughout pregnancy, alcohol intake equal to or exceeding 1 unit per week during pregnancy, back pain, pelvic girdle pain, and neck and shoulder pain during pregnancy, migraine and headache during pregnancy, infections of the genitourinary tract during pregnancy, rheumatoid disorders, use of opioid analgesics during pregnancy, and birth order of the child. Some additional confounding variables were included for birth weight, only (sick-leave of 14 days or longer during pregnancy, use of antiinfectives during pregnancy, and gender of the child), and in addition, some were included for gestational age, only (hospitalisation during pregnancy, vaginal bleeding during pregnancy, and high blood pressure during pregnancy).

### Creation of the propensity score

To create the propensity score, we used logistic regression in which ibuprofen exposure was the dependent variable and the variables considered (i) material or theoretical confounders, or (ii) risk factors for the outcome, were predictors. The propensity score is the predicted exposure status, conditional on measured confounders[34]; it has the advantage of reducing a large number of predictors to a single vector. The final propensity score model included maternal age, parity, birth order, smoking, alcohol intake, pre-pregnancy BMI, education, back pain, pelvic girdle pain, and neck and shoulder pain during pregnancy, migraine and headache during pregnancy, infections of the genitourinary tract during pregnancy, rheumatoid disorders, vaginal bleeding during pregnancy, high blood pressure during pregnancy, and use of opioids during pregnancy. The overlap between the propensity score of the ibuprofen exposed and the NSAIDs unexposed was satisfactory. (S1 Fig)

### Statistical analysis

**Fig 2:**
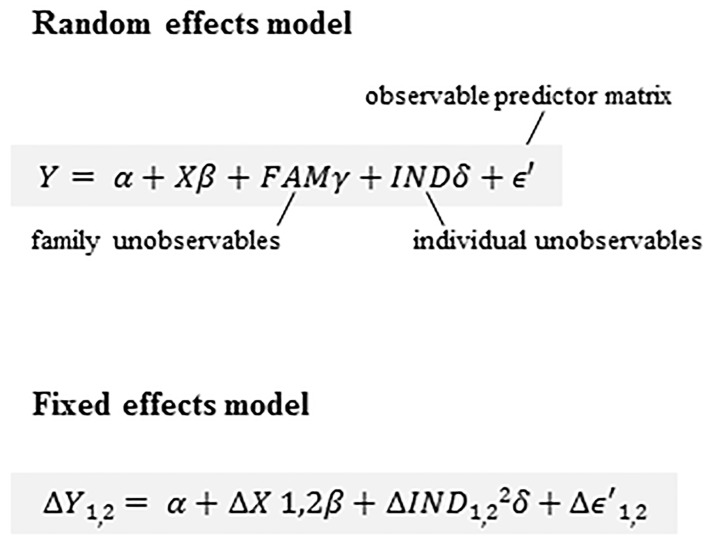
The fixed effects model. The β coefficient gives an estimate of the difference between exposed and non-exposed sibling clusters.

The analyses of the effect of ibuprofen exposure on birth weight and gestational age were carried out in three steps. First, a crude random effects analysis was performed to provide an estimate of the association between ibuprofen exposure during pregnancy and pregnancy outcome for the whole cohort of 28 597 siblings. This estimate is comparable to that obtained from a non-sibling design. Second, the propensity score variable was used as the adjusting variable in the random effects model. Third, a fixed effects model, adjusted for propensity score, was used to adjust for shared unmeasurable family-level effects as well as measured confounders included in the propensity score, see fixed effects formula figure (Fig 2). For all models, the reference category included women who did not report use of NSAIDs during pregnancy. Additional analyses were performed to investigate the association between ibuprofen exposure in only one trimester and ibuprofen exposure in two or more trimesters and the pregnancy outcomes. We also performed a sensitivity analysis on term infants only (infants born in gestational week 37 or thereafter, n = 27 471) to account for the possible effect of gestational age on birth weight.

Statistical analyses were performed with Stata statistical software, version 14.

## Results

Of the 27 904 siblings included in this study, 1080 (3.9%) were prenatally exposed to ibuprofen: 798 were exposed during the first trimester and 481 of these were not exposed to any other trimester; 249 were exposed during the second and/or third trimesters but not in the first, and 33 were exposed anytime during pregnancy without the particular trimester being specified. Regarding duration of use, there were 740 siblings exposed to ibuprofen during any one trimester and 340 siblings exposed to ibuprofen during two or more trimesters.

The characteristics of the mothers who used ibuprofen during pregnancy and mothers who did not use any NSAIDs during pregnancy are presented in Tables 1 and 2. A significantly lower proportion of women using ibuprofen during pregnancy had attained a tertiary level of education and used folic acid supplements prior to and during pregnancy. These women were also more likely to smoke and consume alcohol during pregnancy when compared with the mothers who did not use any NSAIDs during pregnancy (Table 1). Differences were also marked with respect to the women’s health status: a significantly higher proportion of women using ibuprofen during pregnancy suffered from acute and chronic inflammatory and infectious conditions and used analgesic and antiinfective medications during pregnancy when compared with the mothers who did not use any NSAIDs during pregnancy (Table 2).

**Table 1:** Socio-demographic characteristics of the exposed and the unexposed women.

	Ibuprofen exposure anytime during pregnancy		No NSAID exposure during pregnancy	
	N = 1080		N = 26 824	
Maternal age, mean (SD) (years)	29.7 (4.3)		30.0 (4.2)	
BMI, mean (SD) (kg/m^2^)	24.7 (4.7)		23.9 (4.1)	
	n	% of N	n	% of N
Parity				
*0*	462	42.8	10 522	39.2
*1*	420	38.9	11 733	43.7
*>1*	198	18.3^†^	4569	17.0
Marital status with father of child				
*Married/co-habiting*	1041	96.4	26 236	97.8
*Other*	39	3.6^†^	588	2.2
Education				
*Primary*	22	2.0	372	1.4
*Secondary*	351	32.5	6842	25.9
*Tertiary*	691	64.0*	19 169	72.7
Folic acid intake prior to and during pregnancy	447	41.4*	12 766	47.6
Smoking daily at the end of pregnancy	87	8.1*	1058	3.9
Alcohol intake of ≥1 unit per week during pregnancy	123	11.4*	1921	7.2

SD: standard deviation; BMI: Body Mass Index

* Pearson’s χ^2^ test P < 0.001 when compared with no NSAIDs

^†^ Pearson’s χ^2^ test P < 0.01 when compared with no NSAIDs

**Table 2:** Medical characteristics of the exposed and the unexposed women.

	Ibuprofen exposure anytime during pregnancy		No NSAID exposure during pregnancy	
	N = 1080		N = 26 824	
Conditions commonly co-occurring with NSAID therapy during pregnancy	n	% of N	n	% of N
Fever < 38.5°C	734	68.0*	16 850	62.8
Respiratory tract infections	710	65.7^†^	16 405	61.2
Migraine and/or headache	704	65.2*	10 062	37.5
Musculoskeletal pain				
*Back pain*	635	58.8*	13 829	51.5
*Neck and shoulder and abdominal pain*	516	47.8*	9620	35.9
*Pelvic girdle pain*	477	44.2*	10 354	38.6
Genitourinary tract infections	110	10.2^†^	2140	8.0
Rheumatoid disorders	60	5.6*	901	3.4
Co-medication during pregnancy				
Paracetamol	799	74.0*	11 965	44.6
Antiinfectives^a^	173	16.0*	3244	12.1
Opioid analgesics ^b^	53	4.9*	425	1.6
Immunosuppressants ^c^	7	0.6^†^	107	0.4
Other indicators of health during pregnancy				
Nausea	831	76.9	20 347	75.8
Sick-leave > 14 days	384	35.6^‡^	8683	32.4
Antepartum bleeding	120	11.1	2756	10.3
High blood pressure	78	7.2	1704	6.3
Glycosuria	62	5.7	1610	6.0
Hospitalisation	49	4.5	1256	4.7

* Pearson’s χ^2^ test P < 0.001 when compared with no NSAIDs

^†^ Pearson’s χ^2^ test P < 0.01 when compared with no NSAIDs

^‡^ Pearson’s χ^2^ test P < 0.05 when compared with no NSAIDs

^a^ Antiinfectives included antibacterials, antivirals and antimycotics.

^b^ Opioid analgesics included codeine alone and in combination with paracetamol, morphine, tramadol, oxycodone, buprenorphine and dextropropoxyphene.

^c^ Immunosuppressants included mineralocorticoids and glucocorticoids, selective immunosuppressants and TNF-α inhibitors.

The mean birth weights of infants exposed to ibuprofen anytime during pregnancy, during the first trimester only and during the second and/or third trimesters only were 3596 ± 585 grams, 3576 ± 573 grams, and 3660 ± 587 grams, respectively. The mean birth weight of infants born to women who did not use any NSAIDs during pregnancy was 3624 ± 567 grams. There were 31 infants (3.0%) exposed to ibuprofen during pregnancy that were born with a birth weight of less than 2500 grams, as compared to 558 infants (2.1%) who were not exposed to any NSAIDs during pregnancy.

The mean gestational age (39.2 weeks) was the same for all infants whether they were exposed to ibuprofen anytime during pregnancy, during the first trimester only and during the second and/ or third trimesters or not exposed at all. There were 54 infants (5.0%) exposed to ibuprofen during pregnancy that were born before the 37^th^ gestational week, in contrast to 1034 infants (3.8%) who were not exposed to any NSAIDs during pregnancy.

The intra-class correlation within sibling clusters for gestational length and birth weight was 0.40 and 0.21 respectively, indicating a small effect of familial factors.

Tables 3 to 6 show the effect of ibuprofen on birth weight and gestational age. In the propensity score adjusted random effects model, infants exposed to ibuprofen during the first trimester on average weighed 50 grams less than infants whose mothers did not use any NSAIDs during pregnancy (β: -50 grams; 95%CI -94 grams to -7 grams). In the fixed effects model, allowing for adjustment for familial factors, infants exposed to ibuprofen during the first trimester on average weighed 79 grams less than infants whose mothers did not use any NSAIDs during pregnancy (β: -79 grams; 95%CI -133 grams to -25 grams). We did not find any effect of ibuprofen exposure in the second and/or third trimesters only on birth weight (Table 3). Moreover, we did not find any effect of increased duration of exposure on birth weight: in the fixed effects model, infants exposed to ibuprofen during any one trimester on average weighed 61 grams less than infants whose mothers did not use any NSAIDs during pregnancy (β:-61 grams; 95%CI -105 grams to -17 grams) but no significant effect was seen in infants exposed to ibuprofen during two or more trimesters (β:-63 grams; 95%CI -130 grams to 3 grams) (Table 5). The sensitivity analysis on term infants did not change the effect estimates for timing as shown in S1 Table or duration of exposure as shown in S2 Table.

**Table 3:** Associations of ibuprofen with birth weight according to timing of exposure. All values are given in grams.

Exposure during pregnancy	Random effects	Random effects	Fixed effects
	*Crude model*	Adjusted model*	Adjusted model*
	Mean difference (β) (95%CI)	Mean difference (β) (95%CI)	Mean difference (β) (95%CI)
No NSAID exposure	*ref*	*ref*	*ref*
*n = 26 824*			
Anytime	**-41 (-71 to -11)**	**-35 (-65 to -5)**	**-67 (-106 to -29)**
*n = 1080*			
First trimester only	**-54 (-98 to -11)**	**-50 (-94 to -7)**	**-79 (-133 to -25)**
*n = 481*			
Second and/or third trimesters only	24 (-36 to 84)	29 (-31 to 89)	-20 (-94 to 53)
*n = 249*			

The β obtained in the random effects linear regression is representative of the β that would be obtained in a linear regression performed on the whole cohort. The fixed effects linear regression model addresses unmeasured and residual family-level confounding.

* Propensity score variable for ibuprofen exposure during pregnancy included in all models (maternal age, parity, birth order, gender of child, smoking, alcohol intake, pre-pregnancy BMI, education, sick-leave during pregnancy, back pain, pelvic girdle pain, and neck and shoulder pain during pregnancy, migraine and headache during pregnancy, infections of the genitourinary tract during pregnancy, rheumatoid disorders, headache and migraine during pregnancy, use of opioids and antiinfectives during pregnancy).

**Table 4:** Associations of ibuprofen with birth weight according to duration of exposure. All values are given in grams.

Exposure during pregnancy	Random effects	Random effects	Fixed effects
	*Crude model*	Adjusted model*	Adjusted model*
	Mean difference (β) (95%CI)	Mean difference (β) (95%CI)	Mean difference (β) (95%CI)
No NSAID exposure	*ref*	*ref*	*ref*
*n = 26 824*			
During any one trimester	-32 (-67 to 3)	-27 (-62 to 8)	**-61 (-105 to -17)**
*n = 740*			
During any two or more trimesters	**-56 (-108 to -3)**	-47 (-100 to 5)	-63 (-130 to 3)
*n = 340*			

The β obtained in the random effects linear regression is representative of the β that would be obtained in a linear regression performed on the whole cohort. The fixed effects linear regression model addresses unmeasured and residual family-level confounding.

* Propensity score variable for ibuprofen exposure during pregnancy included in all models (maternal age, parity, birth order, gender of child, smoking, alcohol intake, pre-pregnancy BMI, education, sick-leave during pregnancy, back pain, pelvic girdle pain, and neck and shoulder pain during pregnancy, migraine and headache during pregnancy, infections of the genitourinary tract during pregnancy, rheumatoid disorders, headache and migraine during pregnancy, use of opioids and antiinfectives during pregnancy).

**Table 5:** Associations of ibuprofen with gestational age according to timing of exposure. All values are given in days.

Exposure during pregnancy	Random effects	Random effects	Fixed effects
	*Crude model*	Adjusted model*	Adjusted model*
	Mean difference (β) (95%CI)	Mean difference (β) (95%CI)	Mean difference (β) (95%CI)
No NSAID exposure	*ref*	*ref*	*ref*
*n = 26 824*			
Anytime	-0.7 (-1.4 to 0.0)	-0.7 (-1.4 to 0.0)	-1.4 (-2.1 to 0.0)
*n = 1080*			
First trimester only	0.0 (-0.7 to 0.7)	0.0 (-0.7 to 0.7)	-1.4 (-2.8 to 0.0)
*n = 481*			
Second and/or third trimesters only	0.0 (-1.4 to 1.4)	0.0 (-1.4 to 1.4)	-0.7 (-2.8 to 0.7)
*n = 249*			

The β obtained in the random effects linear regression is representative of the β that would be obtained in a linear regression performed on the whole cohort. The fixed effects linear regression model addresses unmeasured and residual family-level confounding.

* Propensity score variable for ibuprofen exposure during pregnancy included in all models (maternal age, parity, birth order, smoking, alcohol intake, pre-pregnancy BMI, education, back pain, pelvic girdle pain, and neck and shoulder pain during pregnancy, migraine and headache during pregnancy, infections of the genitourinary tract during pregnancy, rheumatoid disorders, vaginal bleeding during pregnancy, high blood pressure during pregnancy, use of opioids during pregnancy).

**Table 6:** Associations of extensive ibuprofen exposure with gestational age according to duration of exposure. All values are given in days.

Exposure during pregnancy	Random effects	Random effects	Fixed effects
	*Crude model*	Adjusted model*	Adjusted model*
	Mean difference (β) (95%CI)	Mean difference (β) (95%CI)	Mean difference (β) (95%CI)
No NSAID exposure	*ref*	*ref*	*ref*
*n = 26 824*			
During any one trimester	0.0 (-0.7 to 0.7)	0.0 (-0.7 to 0.7)	-1.4 (-2.1 to 0.0)
*n = 740*			
During any two or more trimesters	-1.4 (-2.8 to 0.0)	-1.4 (-2.8 to 0.0)	-0.7 (-2.8 to 0.7)
*n = 340*			

The β obtained in the random effects linear regression is representative of the β that would be obtained in a linear regression performed on the whole cohort. The fixed effects linear regression model addresses unmeasured and residual family-level confounding.

* Propensity score variable for ibuprofen exposure during pregnancy included in all models (maternal age, parity, birth order, smoking, alcohol intake, pre-pregnancy BMI, education, back pain, pelvic girdle pain, and neck and shoulder pain during pregnancy, migraine and headache during pregnancy, infections of the genitourinary tract during pregnancy, rheumatoid disorders, vaginal bleeding during pregnancy, high blood pressure during pregnancy, use of opioids during pregnancy).

We did not find any association between ibuprofen exposure during pregnancy and gestational age.

## Discussion

The primary finding in our study is that infants exposed to ibuprofen during the first trimester, including those born at term, had a birth weight that is on average 79 grams lower than their siblings not exposed to any NSAIDs during pregnancy.

Our results are similar to those obtained in our previous cohort study[4] where ibuprofen exposure during the second trimester was associated with reduced birth weight. The reason for the discrepancy in timing is likely to be due to the fact that ibuprofen exposure in our cohort study was not trimester-specific, and women who used ibuprofen during the second trimester had also used it in the first. The 79 gram decrease in birth weight, even though statistically significant, is likely to have a negligible effect on the health and development of term and close-to-term infants. However, the scenario could be different in infants born premature. Infants born at gestational week 25 weigh on average 750 grams, so a reduction of 79 grams here would likely be of clinical significance.[35] However, on the basis of the data upon which the present study is based, the reduction in gestational weight might not be the same in absolute terms in those born prematurely as in those born at term. It could well be so that a fixed relative reduction in body weight caused by ibuprofen exposure would be more likely.

We found no association between prenatal ibuprofen exposure and gestational age in the present study, thereby confirming our previous findings and those of the two other studies that assessed the effect of NSAIDs on gestational age.[10, 13]

The birth weight of a neonate is likely to be influenced by factors occurring towards the end of pregnancy. However, recent studies are increasingly pointing to early pregnancy factors that particularly influence placental development, vascularization, and ultimately function.[36] Pregnancy is a state of oxidative stress where increased mitochondrial activity may lead to production of reactive oxidative species (ROS), also in the placenta.[36–38] One hypothesis could be that NSAIDs via possible effects on the mitochondria could create an imbalance in the oxidative state of the placenta, leading to deficiencies in the establishment of blood flow into the intervillous space. Such a disruption in vascularization will influence the transfer of nutrients to the fetus throughout pregnancy.[38–40] The fact that we did not find an association between duration of exposure to ibuprofen and the birth weight or gestational age suggests that timing of exposure (i.e. early in pregnancy) could be a more important factor than the total drug load to the fetus.

Interestingly, the observed effect estimate for birth weight is almost twice as large in our adjusted fixed effects model as the estimate obtained in the adjusted random effects model. There are several possible explanations for this difference. First, it is possible that unmeasured, shared familial confounders obscured the true effect of ibuprofen exposure, and the fixed effects model reduced the effect of these confounders. However, previous research has suggested the sibling studies may produce biased estimates in the case of non-shared confounding between pregnancies.[41] Given that confounding by indication is a strong concern in studies of medication use during pregnancy, and that the indication for ibuprofen use, as well as other familial or maternal characteristics, may change from pregnancy to pregnancy, we cannot rule out bias as a possible alternate explanation for these findings.

Our study has several strengths and weaknesses that merit attention. One strength is that the sibling design enables the adjustment of shared genetic and familial factors across pregnancies–something we were unable to do in our previous cohort study.[28] In addition, using the fixed effects models enabled us to reduce residual confounding due to shared unmeasurable family-level differences.[29] Another strength of our study was the amount of detailed information available both in the MoBa and MBRN. We were able to adjust for maternal socio-demographic factors that are highly related to our outcomes, particularly smoking and alcohol intake during pregnancy. Confounding by indication is unavoidable in studies of drug exposure on pregnancy outcomes but we attempted to reduce this by adjusting for underlying maternal diseases including acute and chronic neuromuscular inflammatory disorders, infections, and co-medication. Finally, the prospective nature of data collection in MoBa reduced the risk of recall bias to a minimum.

The limitations of our study also need to be taken into account. The low participation rate of 40% in MoBa is often a source of concern with respect to possible selection bias, especially with regard to prevalence estimates, as women younger than 25 years, without a life-partner, with a parity >0, and smokers are all under-represented, whereas mothers who take folic acid are over-represented. On the other hand, the fact that only minor differences (below 2% in absolute differences in socio-demographic variables) have been reported between MoBa participants and the general Norwegian population of pregnant women strengthens the representativeness of our material.[30, 31] Since we included sibling births only, our study population may not have been entirely representative of the total population of Norway. Also, our study population size was greatly reduced when compared with the original cohort, and we may have obtained false negative findings. In addition, unshared confounding among siblings leads to more biased estimates than those that would have been obtained in a non-sibling design.[41] Approximately 13% of the siblings were excluded from the study because their mothers lacked information in Qw17 and Qw30. The prevalence of medication use could therefore have been affected but potential associations between ibuprofen exposure and pregnancy outcome would not be expected to change.[42] Finally, we did not have data on dosage of ibuprofen use that would have enabled us to study dose–response effects; however, we created duration of exposure variables as proxies for cumulative dose.

## Conclusion

Our results suggest that prenatal exposure to ibuprofen during the first trimester was associated with a slight decrease in birth weight. The association does not seem to be attributable to shared genetics and family environment and could be explained by either the exposure to ibuprofen or changes in inter-pregnancy factors not accounted for in the analysis. No associations between ibuprofen exposure during pregnancy and gestational age were detected.

## Details of Ethics Approval

The study was approved by the Regional Committee for Ethics in Medical Research, Region South, and the Norwegian Data Inspectorate.

## Supporting Information

S1 Fig Propensity score distribution among the exposed and the unexposed. (TIF)

S1 Table Associations of ibuprofen exposure with birth weight according to timing of exposure limited to term infants. All values are given in grams. The β obtained in the random effects linear regression is representative of the β that would be obtained in a linear regression performed on the whole cohort. The fixed effects linear regression model addresses unmeasured and residual family-level confounding.
* Propensity score variable for ibuprofen exposure during pregnancy included in all models (maternal age, parity, birth order, gender of child, smoking, alcohol intake, pre-pregnancy BMI, education, sick-leave during pregnancy, back pain, pelvic girdle pain, and neck and shoulder pain during pregnancy, migraine and headache during pregnancy, infections of the genitourinary tract during pregnancy, rheumatoid disorders, headache and migraine during pregnancy, use of opioids and antiinfectives during pregnancy).
(DOCX)

S2 Table Associations of ibuprofen exposure with birth weight according to duration of exposure limited to term infants. All values are given in grams. The β obtained in the random effects linear regression is representative of the β that would be obtained in a linear regression performed on the whole cohort. The fixed effects linear regression model addresses unmeasured and residual family-level confounding.
* Propensity score variable for ibuprofen exposure during pregnancy included in all models (maternal age, parity, birth order, gender of child, smoking, alcohol intake, pre-pregnancy BMI, education, sick-leave during pregnancy, back pain, pelvic girdle pain, and neck and shoulder pain during pregnancy, migraine and headache during pregnancy, infections of the genitourinary tract during pregnancy, rheumatoid disorders, headache and migraine during pregnancy, use of opioids and antiinfectives during pregnancy).
(DOCX)
