# Supplementary figures and images for: Association of Prenatal Ibuprofen Exposure with Birth Weight and Gestational Age: A Population-Based Sibling Study
Source: PLoS One. 2016 Dec 9;11(12):e0166971. doi: 10.1371/journal.pone.0166971 (PMC5147859; doi:10.1371/journal.pone.0166971)

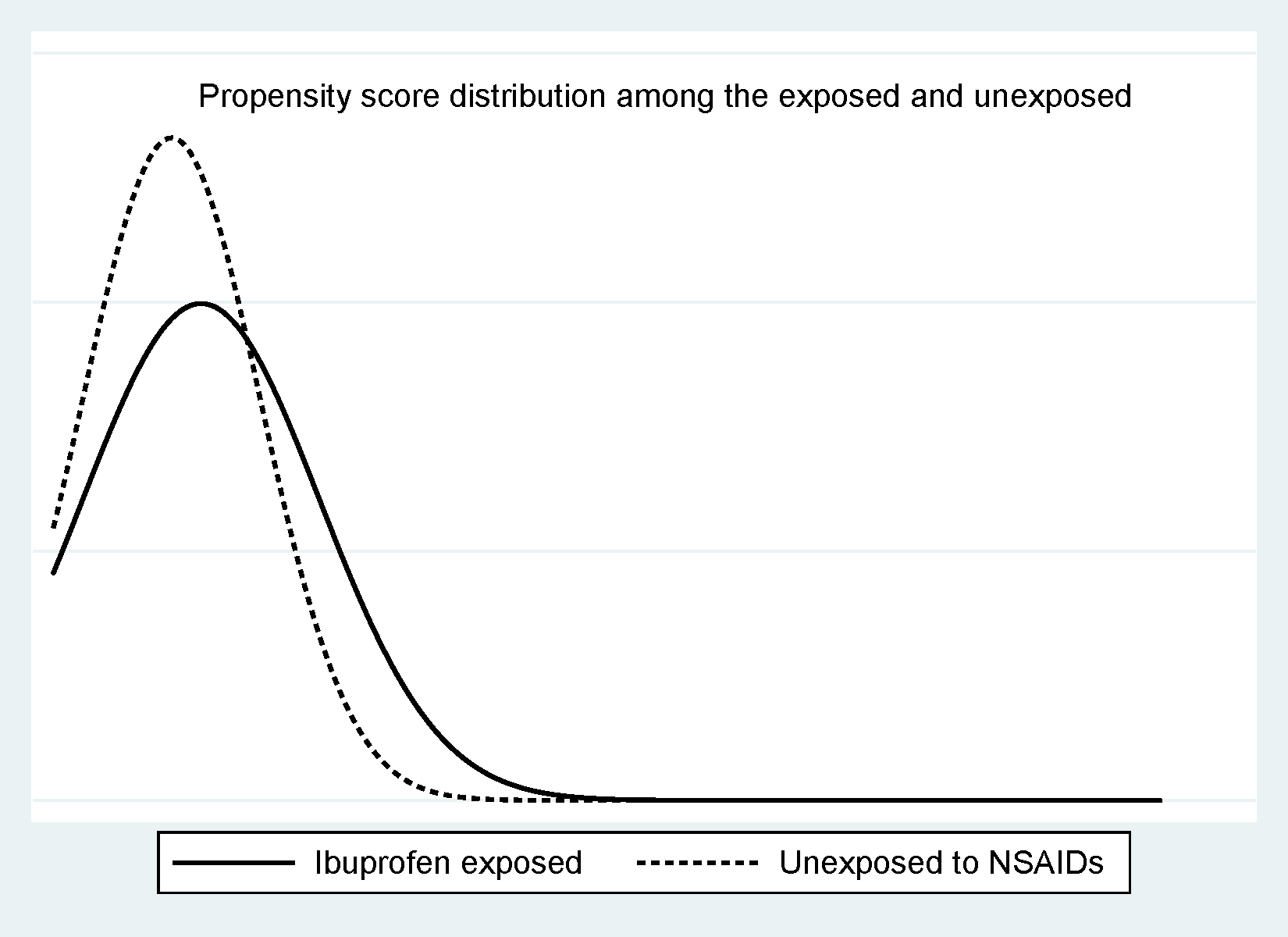

Supplement: S1 Fig — (TIF) [file pone.0166971.s001.tif]
