# Supplementary material for: Association of Prenatal Ibuprofen Exposure with Birth Weight and Gestational Age: A Population-Based Sibling Study
Source: PLoS One. 2016 Dec 9;11(12):e0166971. doi: 10.1371/journal.pone.0166971 (PMC5147859; doi:10.1371/journal.pone.0166971)
Supplement: S1 Table — The β obtained in the random effects linear regression is representative of the β that would be obtained in a linear regression performed on the whole cohort. The fixed effects linear regression model addresses unmeasured and residual family-level confounding. * Propensity score variable for ibuprofen exposure during pregnancy included in all models (maternal age, parity, birth order, gender of child, smoking, alcohol intake, pre-pregnancy BMI, education, sick-leave during pregnancy, back pain, pelvic girdle pain, and neck and shoulder pain during pregnancy, migraine and headache during pregnancy, infections of the genitourinary tract during pregnancy, rheumatoid disorders, headache and migraine during pregnancy, use of opioids and antiinfectives during pregnancy). (DOCX) [file pone.0166971.s002.docx]

S1 Table. Associations of ibuprofen exposure with **birth weight** according to **timing of exposure** limited to **term infants**. All values are given in grams.

| **Exposure during pregnancy** | Random effects  *Crude model*  Mean difference (β) (95%CI) | Random effects  *Adjusted model^*^*  Mean difference (β) (95%CI) | Fixed effects  *Adjusted model^*^*  Mean difference (β) (95%CI) |
| --- | --- | --- | --- |
| No NSAID exposure | *ref* | *ref* | *ref* |
| *n = 25 790* |  |  |  |
| Anytime | **-28 (-56 to -1)** | -24 (-51 to 3) | **-57 (-92 to -22)** |
| *n = 1026* |  |  |  |
| First trimester only | **-50 (-89 to -10)** | **-47 (-86 to -7)** | **-69 (-119 to -20)** |
| *n = 461* |  |  |  |
| Second and/ or third trimesters only | 36 (-19 to 90) | 40 (-15 to 94) | 4 (-62 to 71) |
| *n = 241* |  |  |  |

The β obtained in the random effects linear regression is representative of the β that would be obtained in a linear regression performed on the whole cohort. The fixed effects linear regression model addresses unmeasured and residual family-level confounding.

*^*^* Propensity score variable for Ibuprofen exposure during pregnancy included in all models (maternal age, parity, birth order, gender of child, smoking, alcohol intake, pre-pregnancy BMI, education, sick-leave during pregnancy, back pain, pelvic girdle pain, and neck and shoulder pain during pregnancy, migraine and headache during pregnancy, infections of the genitourinary tract during pregnancy, rheumatoid disorders, headache and migraine during pregnancy, use of opioids and antiinfectives during pregnancy).
